# Evaluation of 15 Functional Candidate Genes for Association with Chronic Otitis Media with Effusion and/or Recurrent Otitis Media (COME/ROM)

**DOI:** 10.1371/journal.pone.0022297

**Published:** 2011-08-16

**Authors:** Michèle M. Sale, Wei-Min Chen, Daniel E. Weeks, Josyf C. Mychaleckyj, Xuanlin Hou, Miranda Marion, Fernando Segade, Margaretha L. Casselbrant, Ellen M. Mandel, Robert E. Ferrell, Stephen S. Rich, Kathleen A. Daly

**Affiliations:** 1 Center for Public Health Genomics, University of Virginia, Charlottesville, Virginia, United States of America; 2 Department of Medicine, University of Virginia, Charlottesville, Virginia, United States of America; 3 Department of Biochemistry and Molecular Genetics, University of Virginia, Charlottesville, Virginia, United States of America; 4 Department of Public Health Sciences, University of Virginia, Charlottesville, Virginia, United States of America; 5 Department of Human Genetics, Graduate School of Public Health, University of Pittsburgh, Pittsburgh, Pennsylvania, United States of America; 6 Department of Biostatistics, Graduate School of Public Health, University of Pittsburgh, Pittsburgh, Pennsylvania, United States of America; 7 Department of Biostatistical Sciences, Wake Forest University School of Medicine, Winston-Salem, North Carolina, United States of America; 8 Department of Anatomy and Cell Biology, University of Pennsylvania, Philadelphia, Pennsylvania, United States of America; 9 Department of Otolaryngology, Children's Hospital of Pittsburgh of UPMC, Pittsburgh, Pennsylvania, United States of America; 10 Department of Otolaryngology, University of Minnesota, Minneapolis, Minnesota, United States of America; Ohio State University Medical Center, United States of America

## Abstract

DNA sequence variants in genes involved in the innate immune response and secondary response to infection may confer susceptibility to chronic otitis media with effusion and/or recurrent otitis media (COME/ROM). We evaluated single nucleotide polymorphisms (SNPs) in 15 functional candidate genes. A total of 99 SNPs were successfully genotyped on the Sequenom platform in 142 families (618 subjects) from the Minnesota COME/ROM Family Study. Data were analyzed for association with COME/ROM using the Generalized Disequilibrium Test (GDT). Sex and age at exam were adjusted as covariates, relatedness was accounted for, and genotype differences from all phenotypically discordant relative pairs were utilized to measure the evidence of association between COME/ROM and each SNP. SNP rs2735733 in the region of the mucin 5, subtypes A/C gene (*MUC5AC*) exhibited nominal evidence for association with COME/ROM (P = 0.002). Two additional SNPs from this region had P values<0.05. Other variants exhibiting associations with COME/ROM at P<0.05 included the *SCN1B* SNP rs8100085 (P = 0.013), *SFTPD* SNP rs1051246 (P = 0.039) and *TLR4* SNP rs2770146 (P = 0.038). However, none of these associations replicated in an independent sample of COME/ROM families. The candidate gene variants examined do not appear to make a major contribution to COME/ROM susceptibility, despite *a priori* evidence from functional or animal model studies for a role in COME/ROM pathology.

## Introduction

Incidence and prevalence of otitis media (OM) has declined since 2000 when infant immunization with pneumococcal conjugate vaccine was adopted in the United States [1], [2], [3]. However, OM is still a very common childhood disease and remains a major cause of morbidity and hearing loss in young children [4], [5]. Children with an affected sibling are at higher risk, and OM clusters in families across generations [6], [7]. Twin studies in the United States, Norway, England and Wales have reported a high degree of heritability for recurrent and chronic OM [8], [9], [10], defined as more than 3 episodes of acute OM in a year, or middle ear fluid persisting for 3–4 months [11], [12].

Our previous research on genetic contributions to chronic otitis media with effusion and/or recurrent otitis media (COME/ROM) include a complex segregation analysis of 173 families suggesting single gene effects [13], a genome-wide linkage scan in 121 families providing support for loci on chromosomes 10q and 19q [14], and an investigation of the human ortholog (*FBXO11*) of the deaf mouse mutant *jeff* gene, a single gene model of chronic/recurrent OM in our family-based study [15].

We selected 15 candidate genes involved in the innate immune response or the ability to clear infectious agents implicated in OM, and evaluated common variation across the coding regions of these genes for association with COME/ROM.

## Methods

### Ethics statement

This study was conducted under Institutional Review Board approval at the University of Minnesota, Wake Forest University, and University of Pittsburgh, and adhered to the tenets of the Declaration of Helsinki. Written consent was provided by all participants or, in the case of children, by their parent or legal guardian.

### Subjects

Subjects who had tympanostomy tube surgery for COME/ROM (probands) and their families were recruited for the study, which has been described previously [14], [15], [16]. All probands were treated with tympanostomy tubes. Participating first and second degree relatives and parents of probands were classified as affected or unaffected based on 1) treatment with tympanostormy tubes, or 2) four data sources. Participants or parents completed a history form documenting history and risk factors for COME/ROM. The study otolaryngologist performed an ear examination to determine presence of OM sequelae without knowledge of the subject's prior OM history, an audiologist conducted tympanometric testing at three frequencies (226, 630 or 710, and 1400) to detect abnormal middle ear mechanics, and medical records were abstracted to document tympanic membrane and middle ear findings consistent with COME/ROM, and duration of OME. Hearing was screened at 20 dB for speech frequencies. Phenotype information comes from four sources: self or parent reported OM history, ear examination, tympanometric findings, and medical record abstract (see Material S1). Affected status of probands' relatives was based on having positive findings from at least two of these four sources (Table S1). A total of 142 families with at least two individuals with the diagnosis of COME/ROM were used in the candidate gene analyses.

We carried out a replication analysis in an independent study of otitis media [17] that consisted of 1,583 genotyped individuals from 441 Caucasian families. In order to assure a history of significant ear disease, two or more full siblings who both or all had undergone tympanostomy tube insertion were enrolled. The need for tympanostomy tube insertion established that the subject's history of middle ear disease was truly significant, resulting in the need for a surgical procedure. A subject was only considered “affected” if he/she had undergone tympanostomy tube insertion at least once for recurrent/persistent OM, while a subject was considered “unaffected” if he/she had never had tympanostomy tubes and had no known history of recurrent/persistent OM. The remaining subjects were considered as having “unknown” disease status. Otoscopic examinations and tympanograms were conducted at at entry if requested by the parent for patient information not study data since the condition of the ears at entry did not determine eligibility and the tubes may have been inserted many years prior to study entry.

### SNP selection and genotyping

DNA was isolated from blood using the Gentra PureGene method (Qiagen, Valencia CA). Candidate genes were selected on the basis of a role in innate host defense and acute inflammatory response or chronic response and effusion, and prior evidence in the literature for involvement in OM. We selected the largest known isoform, added 5 kb upstream and downstream of the coding region, and used HapMap CEU (Centre d'Etude Polymorphisme Human) data (HapMap Data Rel#21/phaseII Jul06, on NBCI B35 assembly, dbSNP b125) to identify SNPs with minor allele frequency (MAF)≥0.05. For mucin 5, subtypes A/C (*MUC5AC*), we used the combined largest mRNA sequence (AF015521; AJ298317; AF043909), which encompasses *MUC5AC* NC_000011.8. We used the pairwise tagging option of Tagger [18] (r^2^>0.8) to select the miminal tagSNP set for each gene. Genotyping was conducted on the Sequenom platform using the iPlex assay [19]. A total of 99 SNPs were successfully genotyped in >95% of samples (see Table 1 for gene summary). Concordance rates were calculated on the basis of 21 replicates included for genotyping on the Sequenom platform at the same time that all samples were assayed. Fifty-four SNPs were 100% concordant; 19 SNPs had one discordant call; 25 SNPs had two discordant calls; and 1 SNP (rs778588 near CD14) had three discordant calls. The concordance rates for nominally associated SNPs were 100% (rs1051246, rs7396030, rs2075859, rs8100085) or 95% (rs2735733, rs2770146; equivalent to one discordant call out of 21 or 20 replicate pairs respectively).

**Table 1:** Selected candidate genes and number of SNPs genotyped.

Candidate gene	Gene symbol	Chromo-some	Gene size* (kb)	Region genotyped (kb)	Total no. SNPs in region†	No. SNPs successfully genotyped	SNPs captured at r^2^>0.8		Mean r^2^
							N	%	
β-defensin 1	DEFB1	8	7.4	13.0	69	10	51	74%	0.982
CD14 antigen precursor	CD14	5	1.9	9.8	9	4	9	100%	0.963
Ecotropic viral integration site 1	EVI1	3	61.5	57.3	73	12	73	100%	0.978
Interleukin 1-β	IL1B	2	7.0	9.1	10	3	8	80%	0.952
Interleukin 8	IL8	4	3.2	n/a	4	1	4	100%	0.983
Interleukin 10	IL10	1	4.9	7.8	21	5	9	43%	0.977
Lactotransferrin	LTF	3	28.9	36.3	33	7	29	88%	0.966
Mannose-binding lectin precursor	MBL2	10	6.3	13.5	46	10	46	100%	0.965
Mucin 2	MUC2	11	29.5	35.3	26	13	22	85%	0.990
Mucin 5, subtypes A/C	MUC5AC	11	75.8	27.1	18	8	13	72%	0.968
Sodium channel, voltage-gated, type I-β	SCN1B	19	9.8	n/a	1	1	1	100%	1.000
Surfactant Protein A1	SFTPA1	10	3.3	n/a	1	1	1	100%	1.000
Surfactant Protein D	SFTPD	10	11.4	17.0	40	8	12	30%	1.000
Toll-like receptor 2	TLR2	4	21.8	23.1	12	8	10	83%	0.983
Toll-like receptor 4	TLR4	9	13.2	17.3	30	8	21	70%	0.951
**Total**			**285.9**	**266.6**	**393**	**99**	**309**	**77%**	**0.916**

*Largest isoform.

†CEU population, minor allele frequency (MAF)>0.05, largest isoform of gene plus 5 kb upstream and downstream, based on HapMap data rel #24 phase II/Nov 08, on NCBI B36 assembly, dbSNP B126.

Candidate genes selected on the basis of their function in innate host defense and acute inflammatory response included Toll-like receptor 4 (*TLR4*) [20], Toll-like receptor 2 (*TLR2*) [21], [22], Beta-defensin 1 (*DEFB1*) [23], Surfactant protein A1 (*SFTPA1*) [24], [25], [26], Surfactant protein D (*SFTPD*) [27], [28], Interleukin-8 (*IL8*) [29], [30], [31], Interleukin-1 β (*IL1B*) [31], Interleukin 10 (*IL10*) [20], and Lactotransferrin (*LTF*) [32]. Genes selected for their role in chronic response and effusion were mucin 2 (*MUC2*) [33], mucin 5, subtypes A/C (*MUC5AC*) [34], Mannose-binding lectin precursor (*MBL2*) [35], CD14 antigen precursor (*CD14*) [36], Sodium channel, voltage-gated, type I-β (*SCN1B*) [37], and Ecotropic viral integration site 1 (*EVI1*) [38].

### Statistical analyses

Deviations from Hardy Weinberg equilibrium (HWE) in unaffected founder individuals were determined using the exact test [39]. Four SNPs with small HWE P values were excluded from the analysis and the remaining 99 SNPs have P>0.001. Data were checked for Mendelian errors using PedCheck [40] and pedstats [39] and poorly performing SNPs or a minimal dataset required to resolve errors were removed from analyses. Two monozygotic (MZ) twin pairs were detected and incorporated in the association analysis.

Data were analyzed for association with COME/ROM using the Generalized Disequilibrium Test (GDT) [41]. The GDT utilizes the genotype differences of all phenotypically discordant relative pairs in assessing association within families. This test has been shown to be consistently more powerful than PDT [42], FBAT [43] and several other family-based association tests for a common disease [41]. It allows modeling of covariates and IBD allele sharing, and information from extended pedigrees is efficiently used without breaking extended pedigrees into multiple nuclear families, as implemented in other methods. Compared to other association tests that also examine between-family association, the GDT method has the advantage of protecting from population stratification between families, and being less affected by genetic heterogeneity from multiple susceptibility genes. In GDT analyses, we modeled sex and age at exam (considered a proxy for generation/clinical practice) as covariates. Identity-by-descent (IBD) statistics were estimated using the Merlin package [44], and were incorporated in the GDT statistics.

## Results

### Population characteristics

The genotyped population included 618 individuals from 142 families (Table 2). One hundred thirty-two families were non-Hispanic and of European ancestry. The 10 non-European families consisted of seven families that described themselves as non-Hispanic and of mixed race, one mixed race Hispanic family, one Asian family, and one Native American family. As family-based tests of association using the GDT are robust to population stratification between families [41], all families were retained for analyses.

**Table 2:** Participant characteristics.

Trait	Value (affected, unaffected)
Number of families	142
Number of subjects	618
Female	52.8%
Age, mean ± SD	27.1±16.6 (28.2±16.5, 25.4±16.5)
Affected with COME/ROM	61.5%
Caucasian	95.3%
Non-Hispanic	99.0%
Smokers in the home, mean ± SD	0.74±0.85
Attending day care centers	52.4% (50.8%, 55.0%)
Prior breastfeeding	57.7% (53.5%, 63.4%)
Allergies	30.7% (32.5%, 26.7%)

### Associations with COME/ROM

A total of 99 SNPs encompassing 267 kb were successfully genotyped in subjects from the Minnesota COME/ROM Family Study. Age at exam was the factor most significantly associated with COME/ROM. The odds ratio of association between one year increment and COME/ROM was 0.94, with P = 1.2×10^−16^. This strong association suggests the importance of adjusting for the age effect in the candidate region association analysis. Although the sex effect is not statistically significant after adjusting for age, it was retained as a covariate in the candidate region association analyses.

SNP rs2735733 in the *MUC5AC* gene region exhibited nominal evidence (P = 0.002) for association with COME/ROM. At this SNP, the T allele (minor allele frequency (MAF) 0.461) is more frequent in unaffected individuals than in affected individuals, with the allele frequency difference being 0.106. The odds ratio (OR) of the minor allele was estimated to be 0.646, assuming a population prevalence of 0.1. Although mucin 5B (*MUC5B*) was not initially selected for study as an *a priori* candidate, the genomic region genotyped, including the associated region of *MUC5AC*, overlaps with *MUC5B*. In the region of chromosome 11 encompassing *MUC5AC* (*MUC5B*) and *MUC2*, there were 21 SNPs in total genotyped. Two other SNPs in this region had P values<0.05 (P = 0.049 at rs7396030 and P = 0.041 at rs2075859). The largest effect of a single SNP is OR = 1.57 at rs7396030. SNP rs7396030 is in weak LD with rs2075859 and rs2735733, with r^2^ 0.036 and 0.013 respectively (D′ 0.293 and 0.216 respectively); rs2075859 and rs2735733 are in LD, with r^2^ = 0.678 (and D′ = 0.987). Only founders were used in the LD calculation, conducted using PLINK [45]. Haplotype-based GDT analysis (as implemented in GDT [41]) identified association (although not significant) between haplotype CC at rs2075859 and rs2735733 and COME/ROM, with P value 0.0097, which is consistent with the single SNP association results. Further haplotype analysis based on three SNPs does not produce any significant association (smallest P = 0.43).

We identified several other nominal SNP-COME/ROM associations, including the *SCN1B* rs8100085 (P = 0.013), *SFTPD* rs1051246 (P = 0.039), and *TLR4* rs2770146 (P = 0.038; Table 3).

**Table 3:** GDT association results (P<0.05) for COME/ROM.

Chr	SNP	Position	Allele	Frequency	P	δ	OR	Genes
9	rs2770146	117552892	G	0.307	0.038	−0.062	0.736	*TLR4*
10	rs1051246	81687798	C	0.118	0.039	−0.058	0.499	*SFTPD* *
11	rs7396030	1073364	T	0.196	0.049	0.078	1.565	*MUC2*
11	rs2075859	1207064	T	0.374	0.041	−0.067	0.744	*MUC5AC,MUC5B*
11	rs2735733	1218216	T	0.461	0.002	−0.106	0.646	*MUC5AC,MUC5B*
19	rs8100085	40214959	A	0.313	0.013	−0.090	0.636	*SCN1B*

Note: Analyses are adjusted for sex and age at exam. δ is the allele frequency difference between affected and unaffected individuals. The odds ratio (OR) of association is converted from δ and MAF, assuming a population prevalence of 0.1.

*This SNP was selected due to its proximity to COME/ROM candidate gene *SFTPD*, but is located within the mannose-binding protein-A pseudogene (*MBL1P1*) gene.

We also carried out association analysis in families consisting of European American only (eight families were excluded) and observed the same associations. All six associated SNPs listed in Table 3 retain their significance with P values 0.027, 0.027, 0.045, 0.033, 0.002, and 0.022 respectively. An additional SNP (rs2672812) in the *MUC5A/MUC5B* region also had a P value <0.05 (P = 0.042).

We carried out a replication analysis of the six SNPs in Table 3 in an independent study [17]. The association results in the replication study are shown in Table 4. The allele frequencies in two studies are comparable. Only one SNP in the MUC2 region, rs7396030, reached a P-value <0.05 in the replication study (unadjusted P = 0.0075; P = 0.022 after adjusting for sex). However, the risk allele in the replication study is the major allele, opposite from our study. The allele flip could occur either by chance (with a small probability of 0.025), or as a genuine allele flip that is due to complex linkage disequilibrium with the causal variant in the region (Clarke and Cardon, 2010). The most likely explanation is that both results are false positives. In the replication sample, the LD structure across the MUC5AC/MUC5B/MUC2 region was similar to that observed in the University of Minnesota sample. LD between SNPs rs2075859 and rs2735733 was high (r^2^ = 0.652, D′ = 0.988), but LD between these SNPs and MUC2 SNP rs7396030 was low (D′ = 0.022 and 0.034 respectively).

**Table 4:** GDT association results for SNPs in Table 3 genotyped in the Caucasian subjects from the replication sample.

Chr	SNP	Position	Allele	Frequency	P	δ	OR	Genes
9	rs2770146	117552892	G	0.324	0.84	0.006	−0.200	*TLR4*
10	rs1051246	81687798	C	0.129	0.65	−0.012	−0.454	*SFTPD*
11	rs7396030	1073364	C*	0.802	0.022	0.057	2.295	*MUC2*
11	rs2075859	1207064	T	0.358	0.22	−0.042	−1.238	*MUC5AC,MUC5B*
11	rs2735733	1218216	T	0.447	0.11	−0.052	−1.617	*MUC5AC,MUC5B*
19	rs8100085	40214959	A	0.379	0.47	0.012	0.715	*SCN1B*

Note: Analyses are adjusted for sex.

*Opposite to allele reported in Table 3.

## Discussion

We performed an evaluation of 15 functional candidate genes in a well-characterized population of families from the Minnesota COME/ROM Family Study. We utilized available HapMap data and employed an LD-based tagging approach to survey common variation in these genes. In some cases, few tagging SNPs were available in the genic region at the time of SNP selection. The strongest observed associations were with SNPs in the mucin 5 genomic region, encompassing both *MUC2* and *MUC5AC/MUC5B*.

Middle ear inflammation stimulates the production and release of inflammatory mediators, with subsequent upregulation of a suite of mucin genes [46]. Mucins are a family of glycosylated proteins that function as part of the mucociliary transport system within the middle ear, binding pathogens and helping clear cellular debris [46]. *Muc5ac* is upregulated in the OM rat model [47], and is one of the genes expressed in mucoid effusion from patients [48]. Similarly, *muc5b* is a major component of OME effusions in a rabbit model of OME [49], and is present in middle ear secretory cells of patients with COM [50]. Both *MUC5AC* and *MUC5B* are located on chromosome 11, with overlapping coding regions. One study found that a longer transcript of *MUC5AC* – but not variants of *MUC5B* or *MUC2* – was associated with OME [51]. However, recent evidence showed MUC5B to be the predominant mucin identified in middle ear effusions from children with COME [52]. We plan to explore whether SNPs associated with COME/ROM in the present study (or variants in LD with them) influence isoform production or transcript stability.

Less robust associations were observed with SNPs in the *SCN1B*, *SFTPD* and *TLR4* genes. Interestingly, in rats whose ears were inoculated with *Streptococcus pneumoniae*, one of the most common pathogens in otitis media, *scn1b*, *muc2* and *muc5* were among the genes upregulated [37], although polymorphisms of *SCN1B* do not appear to have been investigated in COME/ROM previously. Surfactant protein D (*sftpd*) knockout animals do not clear influenza A viral infections [53]. *SFTPD* polymorphisms in humans have been associated with surfactant protein D assembly, function, and concentration [54], as well as severe respiratory syncytial virus infections [27], a known precipitating factor for OM [55]. In a mouse model of spontaneous OM, an exon 3 missense mutation in *Tlr4* resulted in lipid A/lipopolysaccharide (LPS) endotoxin insenstivity and an inability to clear Gram-negative bacteria such as *Haemophilus influenzae* [56], a bacterium frequent found in COME [57]. Similar defects in LPS response due to *TLR4* mutations have been shown to exist in humans [58].

This study has several strengths and limitations. The primary strength is the family-based study design, permitting an analytical approach robust to population stratification. Our newly developed GDT approach also allowed us to take into account covariates age and sex. The study included a small sample of non-European families, consequently we are unable to draw any conclusions about whether the observed associations are specific to European American populations. Genotyping inaccuracy could potentially have led to false negative results however, as noted in the Methods, the concordance rates for the SNPs shown in Table 3 exceeded 95%. The candidate gene approach is necessarily limited by the need for evidence of a role in OM pathology, thus we are expanding our search for COME/ROM susceptibility alleles to a genome-wide association study.

Epidemiological studies suggest that six episodes of AOM by age six is not uncommon. From among individuals with medical record abstracts (65% of participants), only 8 subjects (7 probands and 1 sibling) had 6 episodes of OM by age 6, but did not have 3 episodes OM in any 12 month period. All 8 had tubes placement surgery and were also positive for a history of COME/ROM by either ear exam or tympanometry measures, so would have been classified as affected even without meeting more stringent ROM criteria. Although both studies used similar phenotype criteria to determine affected status based on reported history of COME/ROM, medical record review, ear examination and tympanograms, for the primary study, the sibling of the proband did not need to have undergone tympanostomy tube insertion. In contrast, in the replication sample, both siblings were required to have tympanostomy tube insertion. Failure to replicate nominal associations may be a consequence of diagnostic differences between samples, although it is probable that initial associations represent false positive results. Although we cannot rule out the possibility of type 2 error due to lack of power in the initial sample, we can conclude that there are no strong effects on COME/ROM risk by common variants genotyped in these genes.

Since we define COME as middle ear fluid that lasts 3 or more months, and ROM as 3 OM episodes in a year, or 6 episodes by age 6, there is considerable overlap in our sample between COME and ROM. It is not unreasonable to hypothesize that inadequate production of mucins may predispose to ROM whereas excessive production of mucins may predispose to COME, or that different loci contribute to one or the other. Often patients with middle ear fluid are prone to acute OM, so COME may predipose to ROM. In the UMN sample there were 143 participants with a history of both COME and ROM. Disentangling this relationship in future analyses would require a larger sample.

Our results suggest that common variants in the selected candidate genes do not appear to make a major contribution to COME/ROM susceptibility, despite strong *a priori* hypotheses for a role of these genes in disease pathology, suggesting agnostic genome-wide analyses of COME/ROM populations may be a more productive approach to identify susceptibility alleles.

## Supporting Information

Table S1 Criteria for Classifying Family Members as Affected with COME/ROM for the University of Minnesota Study.
(DOCX)

Material S1 Criteria for Classifying Family Members as Affected with COME/ROM for the University of Minnesota Study.
(DOCX)
