# Supplementary material for: Evaluation of 15 Functional Candidate Genes for Association with Chronic Otitis Media with Effusion and/or Recurrent Otitis Media (COME/ROM)
Source: PLoS One. 2011 Aug 16;6(8):e22297. doi: 10.1371/journal.pone.0022297 (PMC3156706; doi:10.1371/journal.pone.0022297)
Supplement: Table S1 — Criteria for Classifying Family Members as Affected with COME/ROM for the University of Minnesota Study. (DOCX) [file pone.0022297.s001.docx]

**Table S1 Criteria for Classifying Family Members as Affected with COME/ROM for the University of Minnesota Study**

| **Data source** | **Abnormal (evidence of ROM/COME history)*** |
| --- | --- |
| Ear exam | Retraction Perforation  Atrophy Drainage  Tympanosclerosis Tympanostomy tube  Cholesteatoma Monomer |
| Tympanogram | **Age >10 yrs** (a)  Y_1_ ≤0.3 or >1.7 mmho (b)  TW_2_ ≥180 daPa (b)  Resonant frequency <630 Hz or >1400 Hz  Abnormal van Huyse pattern at 630 or 1400 Hz |
|  | **Age ≤10 yrs** (c)  Y_1_ ≤0.3 or ≥1.1 mmho  TW_2_ ≥150 daPa  Resonant frequency <710 Hz or >1400 Hz  Abnormal van Huyse pattern at 710 or 1400 Hz |
| Self-reported history  Medical record | **Adult**  Had tympanostomy tubes  ≥3 OM episodes in a year or 6 episodes before age 6  History of perforation, otorrhea, tympanosclerosis, mastoidectomy, cholesteatoma, tympanoplasty.  **Child**  Had tympanostomy tubes  ≥3 OM episodes in a year or 6 episodes before age 6  Tympanostomy tubes Otorrhea  OME => 2 months Perforation  Tympanosclerosis Mastoidectomy  Cholesteatoma Tympanoplasty  => 3 OM episodes in a year or 6 episodes by age 6 |

* A data source was considered abnormal if ≥1 finding was present.

(a): <5th or 95th percentile ranges for adults [[1](#_ENREF_1)]

(b): Y_1_ = static admittance, TW_2_ = tympanometric width.

(c): <5th or >95th percentile ranges for children [[2](#_ENREF_2),[3](#_ENREF_3),[4](#_ENREF_4)]

**Reference**

1. Margolis RH, Goycoolea HG (1993) Multifrequency tympanometry in normal adults. Ear Hear 14: 408-413.

2. Koebsell KA, Margolis RH (1986) Tympanometric gradient measured from normal preschool children. Audiology 25: 149-157.

3. Margolis RH, Heller JW (1987) Screening tympanometry: criteria for medical referral. Audiology 26: 197-208.

4. Margolis RH, Hunter LL, Rykken JR, Giebink GS (1993) Effects of otitis media on extended high-frequency hearing in children. Ann Otol Rhinol Laryngol 102: 1-5.
