# Supplementary material for: Evaluation of 15 Functional Candidate Genes for Association with Chronic Otitis Media with Effusion and/or Recurrent Otitis Media (COME/ROM)
Source: PLoS One. 2011 Aug 16;6(8):e22297. doi: 10.1371/journal.pone.0022297 (PMC3156706; doi:10.1371/journal.pone.0022297)
Supplement: Material S1 — Criteria for Classifying Family Members as Affected with COME/ROM for the University of Minnesota Study. (DOCX) [file pone.0022297.s002.docx]

**Material S1**

**Criteria for Classifying Family Members as Affected with COME/ROM for the University of Minnesota Study**

Full siblings, two or more, who had a history of tympanostomy tube insertion due to a significant history of OM, their parent(s) and other full sibling(s) with no history of tympanostomy tube insertion were eligible for the study. The subjects were selected from patients who presented to the ENT Research Center from several sources: the ENT Clinic at the CHP and satellite clinics, subjects who were in other studies at the ENT Research Center, or from physician- or self-referral. In order to assure a history of significant ear disease, two or more full siblings who both or all had undergone tympanostomy tube insertion were enrolled. A subject was only considered "affected" if he/she had undergone tympanostomy tube insertion at least once for recurrent/persistent OM, while a subject was considered "unaffected" if he/she had never had tympanostomy tubes and had no known history of recurrent/persistent OM. The remaining subjects were considered as having "unknown" disease status. A detailed history regarding recurrent/persistent OM was obtained for each enrolled family member, as well as history regarding risk factors such as breast-feeding, day care attendance, siblings, and exposure to smoking for the enrolled children. Medical records of enrolled children were obtained for review whenever possible. When feasible, children and parent(s) had an ear examination using pneumatic otoscopy by the study physicians (MC and EM). Tympanograms were obtained using a GSI-38 middle ear analyzer (Lucas-Grason-Stadler, INC).
